# Indocyanine green nebulization visualizes the pulmonary bronchus during video-assisted thoracoscopic surgery

**DOI:** 10.1186/s13019-024-03130-x

**Published:** 2025-02-01

**Authors:** Hao Xu, Xun Wu, Songjing Zhao, Zhenfan Wang, Guanchao Jiang, Yun Li, Jian Zhou

**Affiliations:** 1 Department of Thoracic Surgery, Peking University People’s Hospital, Beijing, 100044 China; 2 Department of Thoracic Surgery, Beijing Aerospace General Hospital, Beijing, China; 3 Peking University Health Science Center, Hai Dian Qu, China

**Keywords:** Lung cancer, Thoracoscopic surgery, Indocyanine green nebulization, Near-infrared fluorescence, Pulmonary bronchus visualization, Intraoperative tracheobronchial injury

## Abstract

**Background:**

Intraoperative tracheobronchial injury is a rare but serious complication of lung surgery. With the increasing number of segmentectomies, surgeons need to locate finer and less easily identified segmental bronchi or even subsegmental bronchi. However, there is no simple or feasible method for visualizing the bronchus during surgery.

**Case presentation:**

Herein, we report a case in which indocyanine green (ICG) inhalation was used to visualize the pulmonary bronchus during video-assisted thoracoscopic surgery. The patient was a woman with a GGO located in the anterior segment of the right upper lobe, and thoracoscopic segmentectomy was scheduled. ICG (3.75 mg/ml) was inhaled into the lung on the operative side after single-lung ventilation for 5 min. During surgery, the anterior segmental bronchus was difficult to locate accurately. Under the overlay imaging window of the NIF imaging system, the bronchus was shown in green, indicating the bronchi in contrast to the surrounding lung tissue. We dissected the bronchi with the assistance of fluorescence imaging and were surprised to find that the bifurcation of the anterior and apical bronchi could be clearly identified by navigation via the inhaled ICG and NIF system. Segmentectomy was successfully performed, and no adverse events were recorded.

**Conclusion:**

This case showed that ICG nebulization is feasible and safe for visualizing the pulmonary bronchus during thoracoscopic surgery. This method has great application potential for reducing intraoperative tracheobronchial injury.

## Background

Intraoperative tracheobronchial injury is a rare but serious complication in lung and esophageal surgery that requires early and skilled repair [1–7]. The main cause of intraoperative tracheobronchial injury is anatomical errors, which may include accidental tracheobronchial transection caused by incorrect identification of anatomical structures and accidental bronchial membrane injury caused by dissection of adjacent bronchial structures. Intraoperative tracheobronchial injuries can be recognized directly by the surgeon or by means of a water submersion test and can be repaired by complete anatomic restoration of the lesion with interrupted or running resorbable sutures1 or coverage of the lesion with an intercostal muscle flap [8]. If tracheobronchial injury is not recognized during surgery, it may cause infection, hemoptysis, dyspnea, etc.

NIR fluorescence with indocyanine green (ICG) has been used clinically for decades. However, ICG nebulization is a new intraoperative imaging method that has been applied to the localization of pulmonary nodules and the detection of pulmonary air leakage [9–11]. Previous studies have focused more on delivering ICG aerosols to the distal part of the lung tissue and reducing consumption in the bronchus while ignoring the potential application of this technology in tracheobronchial imaging. We unexpectedly found that this method can be used for intraoperative tracheobronchial imaging during surgery and has the potential to reduce bronchial injury during surgery.

Here, we describe a case in which we utilized ICG nebulization to visualize pulmonary bronchi during thoracoscopic segmentectomy. We describe the feasibility and safety of this novel technique. This case was found in a clinical trial (No. ChiCTR2100053708) in which the effectiveness of ICG inhalation for visualizing small lung nodules during thoracoscopic surgery was evaluated, and the fluorescence images of pulmonary nodules in the first near-infrared window and the second near-infrared window were compared; these were approved by our hospital institutional review board. The patient provided informed consent.

## Case presentation

**Fig. 1:**
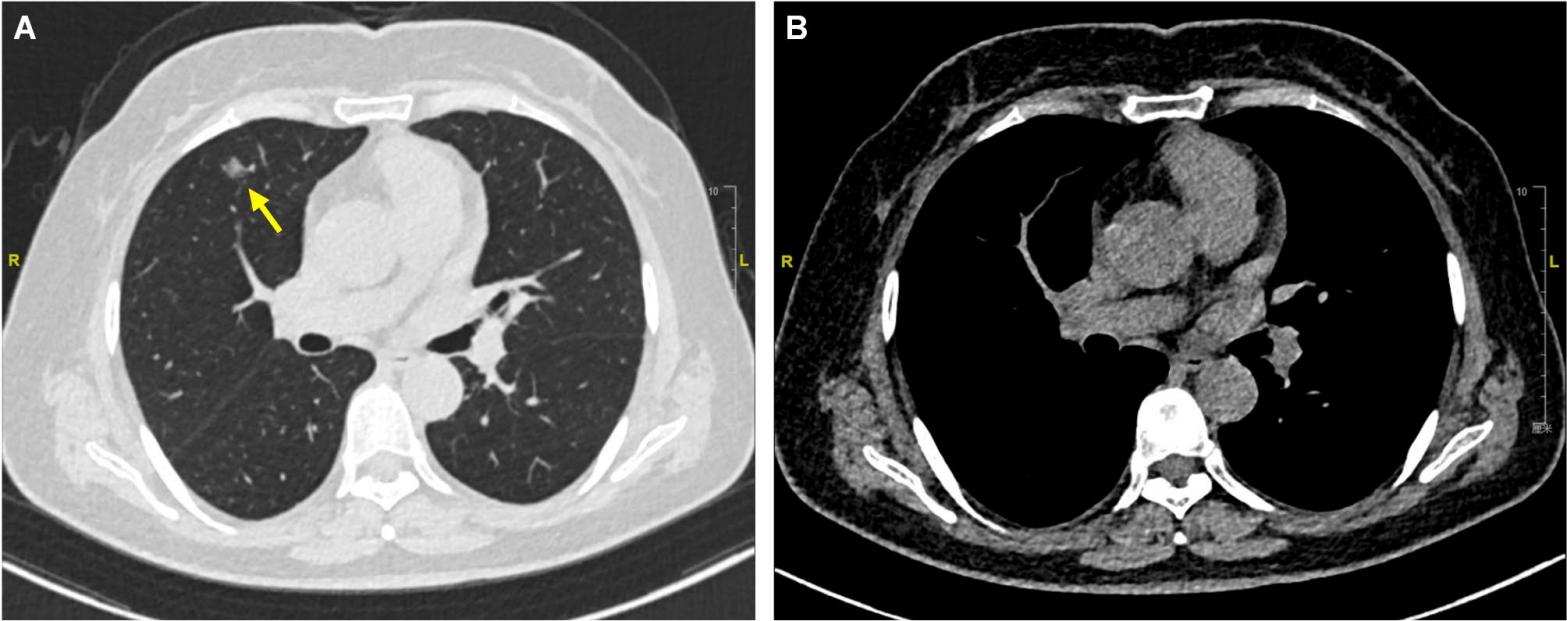
A chest CT revealed a GGO (yellow arrow) in the anterior segment of the right upper lobe (Subfigure **A**). The size of the GGO was approximately 11*9 mm in the lung window, and no visible solid component was observed in the mediastinal window (Subfigure **B**)

The patient was a 58-year-old woman who presented with a chief complaint of cough for 3 months. Upon admission, thin-slice chest computed tomography (CT) was performed and revealed multiple nodules were scattered in two lungs. The largest ground glass opacity (GGO) was located in the anterior segment of the right upper lobe, and the size was approximately 11*9 mm (Fig. 1). The largest nodule was clinically diagnosed as early-stage lung cancer, so thoracoscopic segmentectomy was scheduled.

**Fig. 2:**
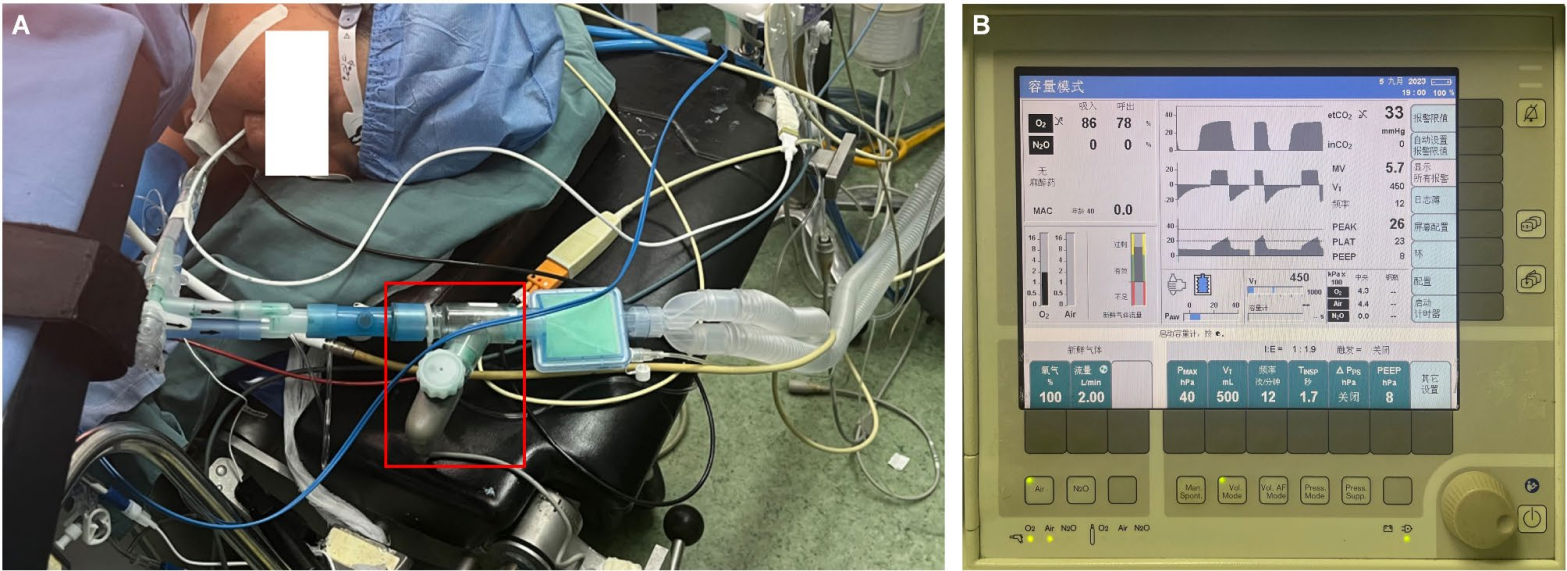
The mesh nebulizer system (red box) was connected between the double-lumen tube and the heat moisture filter of the ventilator (Subfigure **A**). The ventilator was employed in volume-controlled mode, providing a tidal volume of approximately 450–500 ml at 12 respirations per minute, setting the inspiratory-to-expiratory ratio to approximately 1:2 (Subfigure **B**)

After general anesthesia was induced using double-lumen intubation, a mesh nebulizer system (Beijing Winsunny Harmong Science & technology Co. Ltd., Beijing, China) was connected between the double-lumen tube and the heat moisture filter of the ventilator (Fig. 2A). Aerosolized ICG (concentration: 3.75 mg/ml. Yichuang Pharmaceutical Co. Ltd., Dandong, China) was generated by a mesh nebulizer and inhaled into the lung on the operative side after single-lung ventilation. The ventilator was employed in volume-controlled mode, providing a tidal volume of approximately 450–500 ml at 12 respirations per minute, setting the inspiratory-to-expiratory ratio to approximately 1:2 (Fig. 2B). ICG inhalation lasted 5 min.

**Fig. 3:**
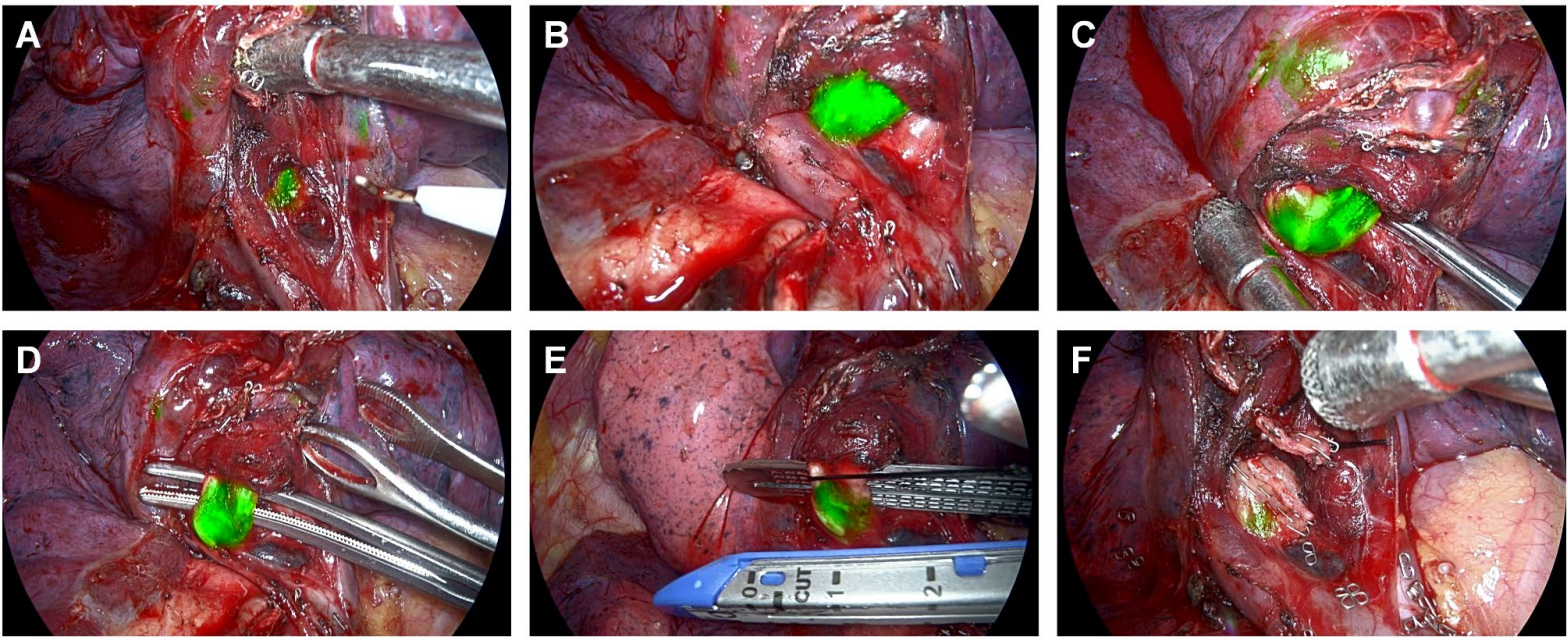
Under the overlay imaging window of the near-infrared fluorescence imaging system, the bronchi were shown in green, which contrasts with the surrounding lung tissue (Subfigures **A** and **B**). More importantly, the bifurcation of the anterior and apical bronchi could be clearly identified (Subfigures **B** and **C**). After the bronchus was fully dissected (Subfigure **D**), it was cut off with an endoscopic blue stapler (Subfigure **E** and **F**)

During surgery, fluorescence imaging of the lung tissue was not obvious (Fig. 3). We first dissected and cut the horizontal fissure and the vein in the anterior segment with endoscopic staplers (EndoGIA). We found it difficult to accurately locate the anterior segmental bronchi, so we hypothesized that using the near-infrared fluorescence (NIF) imaging system and inhaled ICG might help to locate thinner bronchi. Under the overlay imaging window of the NIF imaging system (DPM, Zhuhai Dipu Medical Technology Co., Ltd., Zhuhai, China), the bronchus was shown in green, indicating that the bronchi contrasted with the surrounding lung tissue (Fig. 3A and B). We dissected the bronchi with the assistance of fluorescence imaging and were surprised to find that the bifurcation of the anterior and apical bronchi could be clearly identified by navigation via the inhaled ICG and NIF system (Fig. 3B, C and D). We easily dissected the bronchi without damage. After the bronchus was fully dissected, it was cut off with an endoscopic blue stapler (EndoGIA) (Fig. 3E and F). Other steps were performed according to the standard procedure of our hospital.

The postoperative course was uneventful, and no adverse events were recorded. Pathology of this patient suggested that the nodule was a minimally invasive adenocarcinoma with no metastasis to the lymph nodes. On postoperative day 3, the thoracic drain was removed. The patient was discharged on day 5.

## Discussion and conclusions

We report a case in which aerosolized ICG at a concentration of 3.75 mg/ml was inhaled into the patient’s lung tissue using a nebulizer. Bronchial imaging was performed with an NIF imaging system, which provided significant contrast with the surrounding lung tissue. This method can assist surgeons in identifying bronchi more intuitively during surgery and has clinical application potential for reducing bronchial injury during anatomical processes.

Injuries in the tracheobronchial tree might occur in different ways. Erroneous transections to the bronchi could lead to additional major surgeries and potentially severe complications [1]. Amit Borah et al. reported a case in which the superior segment bronchus of the right lower lobe was erroneously clipped by a surgical clip during basal segmental resection [12]. Notably, when bronchial variation occurs, intraoperative anatomical structure discrimination becomes more difficult [13]. Dissection of adjacent bronchial structures may cause bronchial membrane injury, especially during the process of lymph node dissection. Kawamura et al. reported a case in which the membranous portion of the intermediate bronchus was injured approximately 5 mm in length while dissecting subcarinal lymph nodes [14]. Even though the fistula was closed by knotted suture using 4 − 0 polydioxanone and covered with a pericardial fat pad, postoperative bronchoscopy revealed a slit-like bronchopleural fistula at the intermediate bronchus.

Although preoperative bronchoscopy or CT-based three-dimensional reconstruction are helpful for improving the understanding of patients’ bronchial anatomy, these methods are complicated and cannot provide real-time intraoperative guidance. Intraoperative ICG inhalation for real-time visualization of the bronchus can fill this gap. This method might be useful for surgeons in cases where the bronchi are located deep in the lung parenchyma or where there is anatomic variation in the bronchial tree. In addition, selective transbronchoscopic ICG injection is also an effective bronchography method, but ICG inhalation does not require the assistance of a bronchoscope, is more convenient to perform, causes less damage to the patient’s airway, and is less expensive (approximately $20). Both of these methods are effective bronchography methods, and surgeons can choose their own method according to the actual situation.

According to our experience, as the duration of ICG inhalation increases, lung fluorescence imaging becomes increasingly obvious. This method requires a smaller dose of ICG than does ICG inhalation for the localization of pulmonary nodules, which means that this method results in less stimulation of the airway. If the duration of ICG inhalation is within a short range, only the bronchi will be visualized under the NIF system. The optimal nebulization time for better visualization of different parts of the bronchus should be further studied in the future.

The optimal concentration of inhaled ICG is uncertain. In the study conducted by Wang et al., the concentration of ICG used for inhalation was 3.75 mg/ml, and no adverse events attributed to ICG inhalation were recorded [9]. Therefore, we chose to use an ICG concentration of 3.75 mg/ml. However, there have been no studies on bronchial imaging with ICG inhalation, and the optimal ICG concentration needs to be further verified.

This method requires only ICG drugs and inhalation devices, both of which are inexpensive and readily available, costing approximately $20 in total. Since this case was discovered in a clinical trial described above, all the costs were borne by the researchers, so there was no increase in the hospitalization costs of the patients. In this case, the duration of ICG inhalation was only 5 min, which did not significantly extend the total duration of the operation. In addition, the fluorescence of the bronchus can be displayed in real time in the NIF imaging system, which does not cause inconvenience during the operation.

In this case, no adverse events related to ICG inhalation occurred. However, the effect of this method on breathing during inhalation and its long-term effects are not yet clear. We are currently conducting a clinical trial to verify the feasibility, efficacy and safety of this method, and the results of the study will be reported in a timely manner. With the increasing number of segmentectomies, surgeons need to locate finer and less easily identified segmental bronchi or even subsegmental bronchi. Therefore, we believe that this method has great clinical application value.

In summary, we present a case in which real-time visualization of the bronchi was successful after inhalation of aerosolized ICG. This method is feasible, safe, and has clinical application potential to assist complete bronchotomy, which can avoid bronchial fistula and incomplete resection caused by difficulty in bronchial recognition.

## Data Availability

No datasets were generated or analysed during the current study.

## Abbreviations

ICG: Indocyanine green
CT: Computed tomography
GGO: Ground glass opacity
NIF: Near-infrared fluorescence
